# Supplementary figures and images for: Identification of Low- and High-Impact Hemagglutinin Amino Acid Substitutions That Drive Antigenic Drift of Influenza A(H1N1) Viruses
Source: PLoS Pathog. 2016 Apr 8;12(4):e1005526. doi: 10.1371/journal.ppat.1005526 (PMC4825936; doi:10.1371/journal.ppat.1005526)

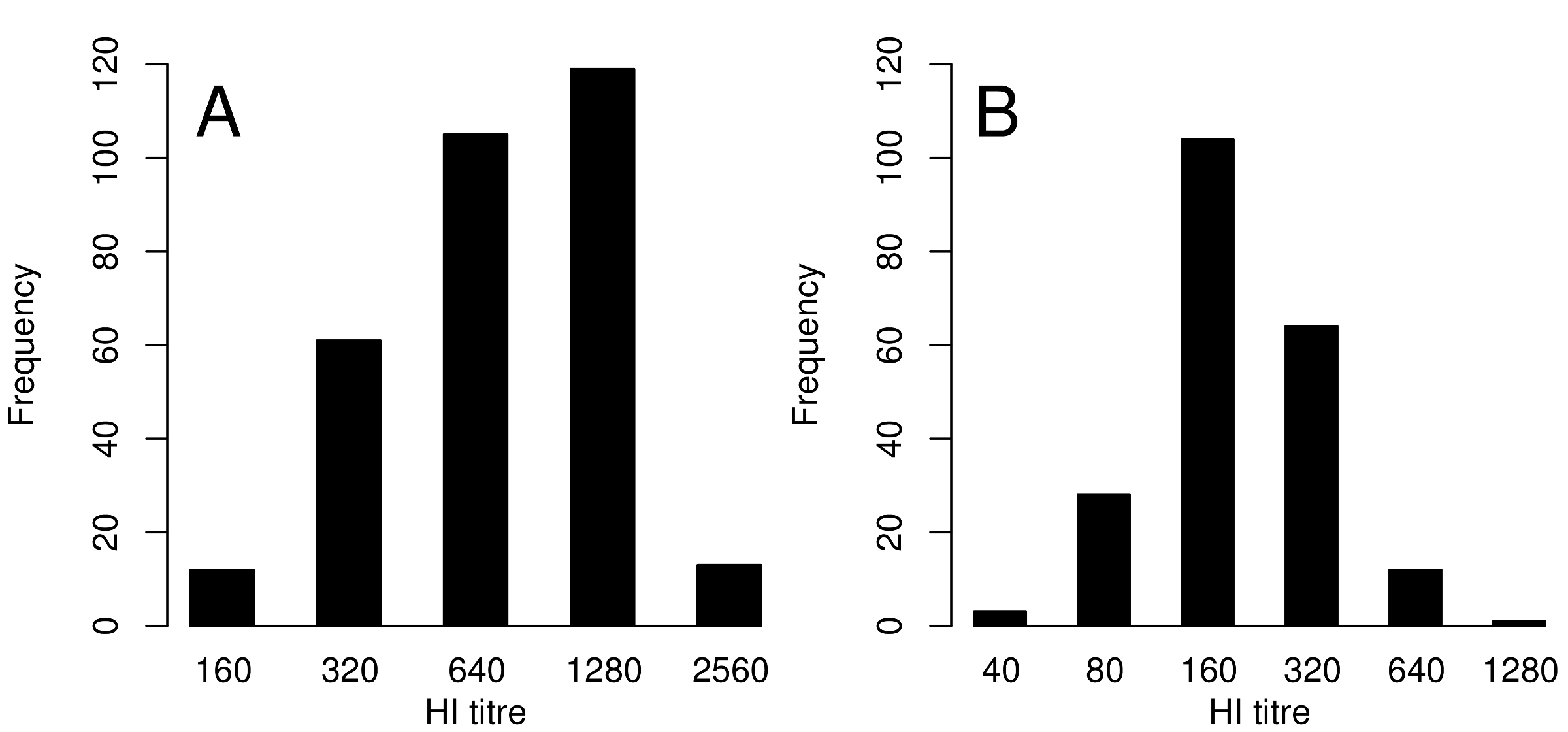

Supplement: S1 Fig — Frequency of HI titers recorded for the virus A/New Caledonia/20/99 tested using antisera raised against A/New Caledonia/20/99 (A: Homologous) and A/Beijing/262/95 (B: Heterologous). (TIF) [file ppat.1005526.s001.tif]

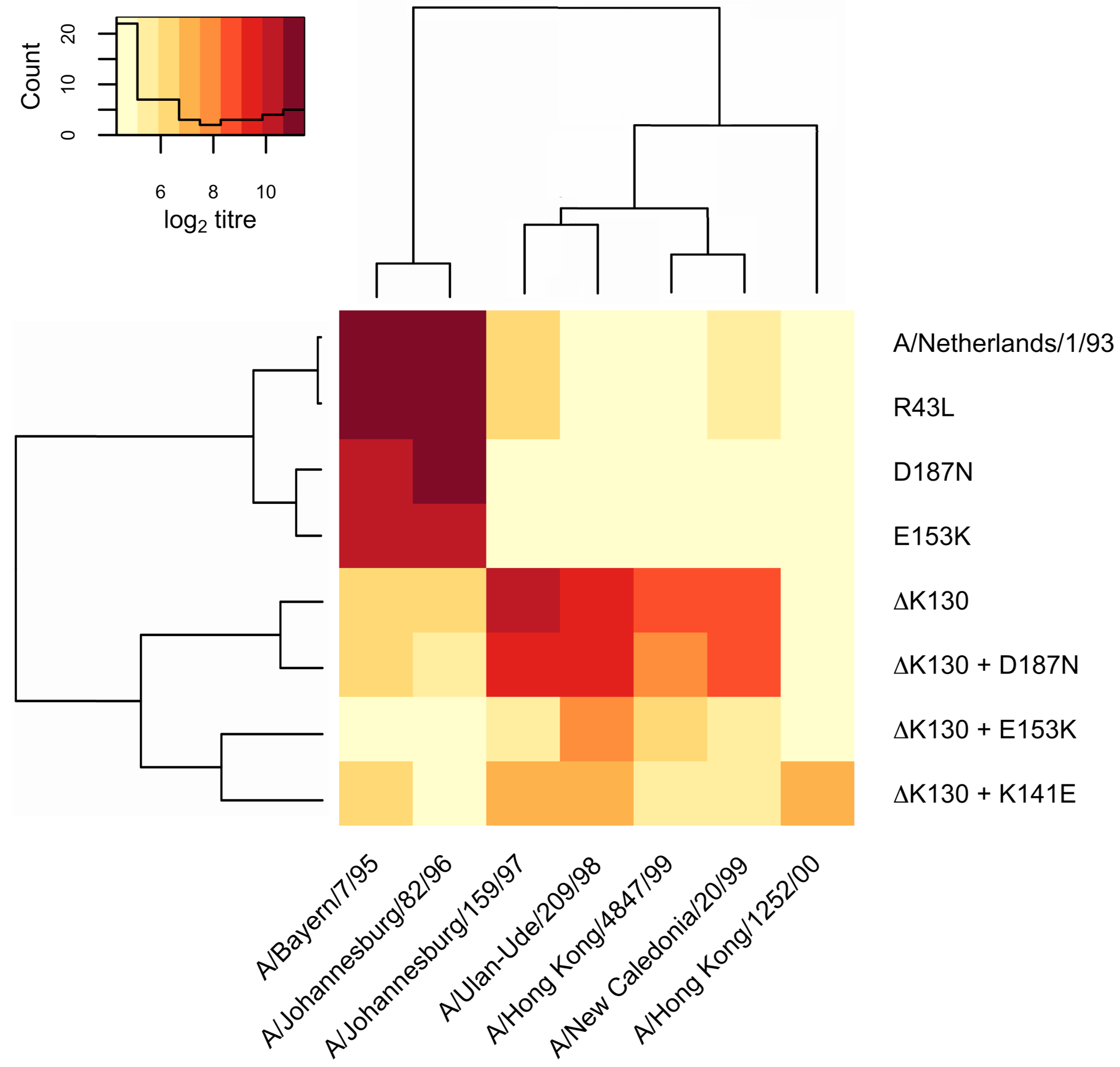

Supplement: S2 Fig — Hierarchical clustering of the wild type Neth93 and mutant viruses generated from it by reverse genetics. Reference strains used to generate antisera are arranged according to their ability to inhibit agglutination of turkey RBCs by each virus. Viruses are simultaneously clustered along the vertical axis according to their antigenic profile. Dendograms indicating antigenic relatedness are shown at the top (for antisera) and to the left (for viruses). Coloring represents log2 HI titer as indicated at top left with the histogram showing the frequency (count) for each titer. (TIF) [file ppat.1005526.s002.tif]

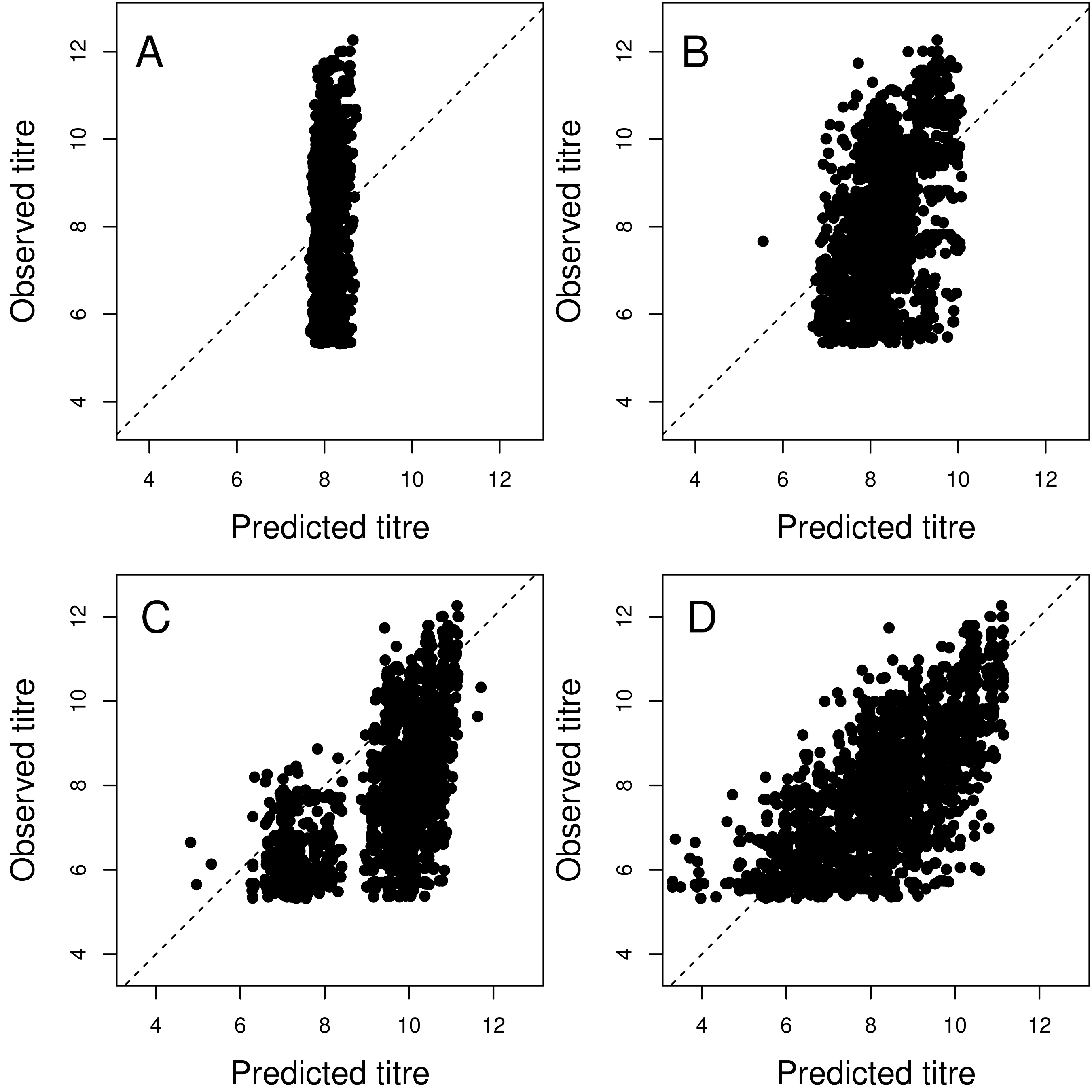

Supplement: S3 Fig — Observed and predicted HI titers plotted on log2 scale (antigenic units) using models trained to data collected prior to a given year and used to predict titres for viruses isolated in that given year. Plots show predictions aggregated across 12 years (1998–2009). Predictive models contained terms for A) The estimated baseline titre B) Average titers for each reference virus, C) Antigenic cluster-defining substitutions ΔK130 and K141E, D) All 18 antigenic substitution(s) shown in Table 1. (TIF) [file ppat.1005526.s003.tif]
