# Supplementary material for: Identification of Low- and High-Impact Hemagglutinin Amino Acid Substitutions That Drive Antigenic Drift of Influenza A(H1N1) Viruses
Source: PLoS Pathog. 2016 Apr 8;12(4):e1005526. doi: 10.1371/journal.ppat.1005526 (PMC4825936; doi:10.1371/journal.ppat.1005526)
Supplement: S1 Table — Models are shown alongside their Δ AIC, the improvement in AIC relative to a null, intercept model (model A). Model terms are consistent with notation used in Eqs 1–7 and are described in full in S2 Table. Models A-H include every combination of base terms introduced in Eq 1. Relative AIC scores led to H being strongly preferred and other combinations were therefore discounted. Models I and J contain 62 branch terms identified using Eq 2. Model J also includes terms for seven substitutions identified using Eq 3. Model K generates a fitted value for every observed combination of reference virus and test virus and corresponds to Eq 7. Models L and M include only substitution terms to explain antigenic differences and no branch terms. Model L includes all 18 identified substitutions (7 identified using Eq 3 and 11 identified using Eq 4). Model M contains terms for the substitutions of highest antigenic impact (K141E and ΔK130) only. (DOCX) [file ppat.1005526.s004.docx]

**S1 Table. Model quality as assessed by AIC.**

| **ID** | **Model** | **Δ AIC** |
| --- | --- | --- |
| A | $k_{0}+\varepsilon_{R}$ | 0 |
| B | $k_{0}+\varepsilon_{D}+\varepsilon_{R}$ | 1,123.24 |
| C | $k_{0}+a_{v}+\varepsilon_{R}$ | 1,820.08 |
| D | $k_{0}+a_{v}+\varepsilon_{D}+\varepsilon_{R}$ | 2,059.26 |
| E | $k_{0}+s_{r}+\varepsilon_{R}$ | 2,298.44 |
| F | $k_{0}+s_{r}+\varepsilon_{D}+\varepsilon_{R}$ | 3,487.73 |
| G | $k_{0}+s_{r}+a_{v}+\varepsilon_{R}$ | 4,082.94 |
| H | $k_{0}+s_{r}+a_{v}+\varepsilon_{D}+\varepsilon_{R}$ | 4,641.86 |
| I | $k_{0}+\sum_{i=1}^{62} m_{i}\delta_{i}(r,v)+s_{r}+a_{v}+\varepsilon_{D}+\varepsilon_{R}$ | 31,610.46 |
| J | $k_{0}+\sum_{j=1}^{7} k_{j}\alpha_{j}\left( r,v \right)+\sum_{i=1}^{62} m_{i}\delta_{i}(r,v)+s_{r}+a_{v}+\varepsilon_{D}+\varepsilon_{R}$ | 34,525.13 |
| K | $k_{0}+s_{r}+a_{v}+\gamma_{rv}+\varepsilon_{D}+\varepsilon_{R}$ | 40,814.98 |
| L | $k_{0}+\sum_{l=1}^{11} k_{l}^{'}\alpha_{l}^{'}\left( r,v \right)+\sum_{j=1}^{7} k_{j}\alpha_{j}\left( r,v \right)+s_{r}+a_{v}+\varepsilon_{D}+\varepsilon_{R}$ | 33,265.94 |
| M | $k_{0}+k_{130}^{'}\alpha_{130}^{'}\left( r,v \right)+k_{141}\alpha_{141}\left( r,v \right)+s_{r}+a_{v}+\varepsilon_{D}+\varepsilon_{R}$ | 26,957.81 |

Models are shown alongside their Δ AIC, the improvement in AIC relative to a null, intercept model (model A). Model terms are consistent with notation used in Equations 1-7 and are described in full in S2 Table. Models A-H include every combination of base terms introduced in Equation 1. Relative AIC scores led to H being strongly preferred and other combinations were therefore discounted. Models I and J contain 62 branch terms identified using Equation 2. Model J also includes terms for seven substitutions identified using Equation 3. Model K generates a fitted value for every observed combination of reference virus and test virus and corresponds to Equation 7. Models L and M include only substitution terms to explain antigenic differences and no branch terms. Model L includes all 18 identified substitutions (7 identified using Equation 3 and 11 identified using Equation 4). Model M contains terms for the substitutions of highest antigenic impact (K141E and ΔK130) only.
