# Supplementary material for: Identification of Low- and High-Impact Hemagglutinin Amino Acid Substitutions That Drive Antigenic Drift of Influenza A(H1N1) Viruses
Source: PLoS Pathog. 2016 Apr 8;12(4):e1005526. doi: 10.1371/journal.ppat.1005526 (PMC4825936; doi:10.1371/journal.ppat.1005526)
Supplement: S2 Table — (DOCX) [file ppat.1005526.s005.docx]

**S2 Table. Reference guide for terms used in S1 Table and in equations throughout the text.**

| Term | Explanation |
| --- | --- |
| *v* | Test virus: each virus tested by HI in compiled antigenic dataset. |
| *r* | Reference virus: each virus used to generate antiserum used to characterise viruses in HI assays in compiled antigenic dataset. |
| *H_r,v_* | HI titer recorded for test virus *v* and antiserum raised against reference virus *r*. |
| *a_v_* | Effect of test virus *v*. Likely indicates variation in receptor-binding avidity. |
| *s_r_* | Effect of reference virus *r*. Likely indicates variation in reference virus immunogenicity or antiserum potency. |
| $\gamma_{rv}$ | Interaction between reference virus *r* and test virus *v.* Represents antigenic relationship between *r* and *v*. |
| $m_{i}\delta_{i}(r,v)$ | Phylogenetic terms: For branch *i* of the phylogenetic tree, $\delta_{i}$ = 1 when the branch lies between test virus *v* and reference virus *r* in a path traced through the tree and $\delta_{i}$ = 0 otherwise. *m_i_* is the effect associated with being positioned on different sides of branch *i* in the tree. |
| $k_{j}\alpha_{j}\left( r,v \right)$ | Substitution terms: For an amino acid difference *j*, $\alpha_{j}$= 1 when the amino acid difference is present between test virus *v* and reference virus *r* and $\alpha_{j}$= 0 otherwise. $k_{j}$ is the effect associated with the presence of the amino acid difference *j.* |
| $k_{l}^{'}\alpha_{l}^{'}\left( r,v \right)$ | Substitution terms: This term is the same as $k_{j}\alpha_{j}\left( r,v \right)$ except that it represents substitutions tested and identified using a model which already included substitutions identified using Equation 3. |
| $m_{\omega}\alpha_{\omega}\left( r,v \right)$ | Substitution terms used for prediction: For an amino acid difference $\omega$ in the set $\Omega$, $\alpha_{\omega}$ = 1 when the amino acid difference is present between test virus *v* and reference virus *r* and $\alpha_{\omega}$= 0 otherwise. $m_{\omega}$ is the effect associated with the presence of the amino acid difference $\omega$*.* |
| $\Omega$ | Set of amino acid differences used for prediction of HI titers. Set includes substitutions identified using both Equation 3 and Equation 4. |
| $\varepsilon_{D}$ | Normally distributed error representing day-to-day variability. |
| $\varepsilon_{R}$ | Residual error. |
| *k*_0_ | Intercept. |
