# Supplementary material for: Identification of Low- and High-Impact Hemagglutinin Amino Acid Substitutions That Drive Antigenic Drift of Influenza A(H1N1) Viruses
Source: PLoS Pathog. 2016 Apr 8;12(4):e1005526. doi: 10.1371/journal.ppat.1005526 (PMC4825936; doi:10.1371/journal.ppat.1005526)
Supplement: S3 Table — Amino acid identity at HA positions 43, 130, 141, 153 and 187 of reference viruses against which antisera were raised and used to antigenically characterize recombinant viruses (* indicates deletion of amino acid corresponding to position 130). Cells are colored according to whether the reference virus against which antiserum was raised lacked or shared each amino acid substitution introduced by mutagenesis to produce recombinant viruses (R43L, ΔK130, K141E, E153K and D187N). Red indicates that the reference virus lacked the substitution introduced into the recombinant virus and so was in the ancestral state (e.g. R43) and blue indicates that the reference virus shared the introduced substitution (e.g. L43). Absence of color indicates that the reference amino acid identity at the position of substitution in the recombinant virus was different from both of the parental viruses (Neth93 and Neth93 Δ130) and from the mutant virus. (DOCX) [file ppat.1005526.s006.docx]

**S3 Table. Antisera used to characterize recombinant viruses.**

| Amino acid substitution resulting from mutagenesis | Reference virus against which antisera were raised | | | | | | |
| --- | --- | --- | --- | --- | --- | --- | --- |
|  | A/Bayern  /7/95 | A/Johannesburg  /82/96 | A/Johannesburg  /159/97 | A/Ulan-Ude  /209/98 | A/Hong Kong  /4847/98 | A/New Caledonia  /20/99 | A/Hong Kong  /1252/2000 |
| R43L | R | R | L | L | L | L | L |
| ΔK130 | K | K | Δ* | Δ* | Δ* | Δ* | Δ* |
| K141E | K | K | K | K | K | K | E |
| E153K | E | E | E | K | G | G | G |
| D187N | D | D | D | D | N | D | D |

Amino acid identity at HA positions 43, 130, 141, 153 and 187 of reference viruses against which antisera were raised and used to antigenically characterize recombinant viruses (* indicates deletion of amino acid corresponding to position 130). Cells are colored according to whether the reference virus against which antiserum was raised lacked or shared each amino acid substitution introduced by mutagenesis to produce recombinant viruses (R43L, ΔK130, K141E, E153K and D187N). Red indicates that the reference virus lacked the substitution introduced into the recombinant virus and so was in the ancestral state (e.g. R43) and blue indicates that the reference virus shared the introduced substitution (e.g. L43). Absence of color indicates that the reference amino acid identity at the position of substitution in the recombinant virus was different from both of the parental viruses (Neth93 and Neth93 Δ130) and from the mutant virus.
