# Supplementary material for: Identification of Low- and High-Impact Hemagglutinin Amino Acid Substitutions That Drive Antigenic Drift of Influenza A(H1N1) Viruses
Source: PLoS Pathog. 2016 Apr 8;12(4):e1005526. doi: 10.1371/journal.ppat.1005526 (PMC4825936; doi:10.1371/journal.ppat.1005526)
Supplement: S4 Table — Geometric mean HI titers are recorded as the reciprocal of the highest dilution of a particular antiserum that inhibited hemagglutination of a standardized concentration of red blood cells by eight hemagglutinating units of each recombinant virus. A visual description of these data is provided in S2 Fig. (DOCX) [file ppat.1005526.s007.docx]

**S4 Table. Mean HI titers for recombinant viruses measured against antisera presented in S1 Table.**

| Antiserum raised against | A/Bayern  /7/95 | A/Johannesburg  /82/96 | A/Johannesburg  /159/97 | A/Ulan-Ude  /209/98 | A/Hong Kong  /4847/98 | A/New Caledonia  /20/99 | A/Hong Kong  /1252/2000 |
| --- | --- | --- | --- | --- | --- | --- | --- |
| Parent virus and recombinant viruses |  |  |  |  |  |  |  |
| A/Netherlands/1/93 (Neth93) | 2792 | 2792 | 87 | 28 | 20 | 40 | 20 |
| Neth93 ΔK130 | 87 | 104 | 1174 | 698 | 453 | 453 | 24 |
| Neth93 R43L | 2792 | 2792 | 80 | 28 | 20 | 40 | 20 |
| Neth93 E153K | 1660 | 1522 | 28 | 34 | 20 | 24 | 20 |
| Neth93 D187N | 1396 | 1660 | 698 | 20 | 20 | 28 | 20 |
| Neth93 ΔK130 K141E | 62 | 24 | 174 | 160 | 48 | 48 | 174 |
| Neth93 ΔK130 E153K | 28 | 20 | 57 | 247 | 80 | 44 | 20 |
| Neth93 ΔK130 D187N | 67 | 52 | 698 | 538 | 293 | 320 | 28 |

Geometric mean HI titers are recorded as the reciprocal of the highest dilution of a particular antiserum that inhibited hemagglutination of a standardized concentration of red blood cells by eight hemagglutinating units of each recombinant virus. A visual description of these data is provided in S2 Fig.
