# Supplementary material for: Identification of Low- and High-Impact Hemagglutinin Amino Acid Substitutions That Drive Antigenic Drift of Influenza A(H1N1) Viruses
Source: PLoS Pathog. 2016 Apr 8;12(4):e1005526. doi: 10.1371/journal.ppat.1005526 (PMC4825936; doi:10.1371/journal.ppat.1005526)
Supplement: S5 Table — * Predictions for this model were made using a parameter for date of test. Date was not used as a predictor in any other model. † This is the upper limit on the lower 95% credible interval for the mean, absolute test error. (DOCX) [file ppat.1005526.s008.docx]

**S5 Table. Average absolute prediction error (antigenic units) across various predictive models.**

| Antigenic substitutions | Antiserum effects | Virus effects | Test error.  Mean (± SEM) | 95% upper limit  on test error^†^ |
| --- | --- | --- | --- | --- |
| None | None | None | 1.48 (± 0.0020) | 3.00 |
| None* | None* | None* | 1.43 (± 0.0028) | 3.01 |
| None | Estimated | None | 1.44 (± 0.0024) | 3.37 |
| None | None | Estimated | 1.53 (± 0.0033) | 3.18 |
| Major | Estimated | None | 1.06 (± 0.0018) | 2.43 |
| Major | Estimated | Estimated | 0.82 (± 0.0016) | 1.87 |
| All | Estimated | None | 0.75 (± 0.0013) | 1.74 |
| All | Estimated | Estimated | 0.65 (± 0.0012) | 1.63 |

* Predictions for this model were made using a parameter for date of test. Date was not used as a predictor in any other model.

^†^ This is the upper limit on the lower 95% credible interval for the mean, absolute test error.
